# Rumor Diffusion in an Interests-Based Dynamic Social Network

**DOI:** 10.1155/2013/824505

**Published:** 2013-12-21

**Authors:** Mingsheng Tang, Xinjun Mao, Zahia Guessoum, Huiping Zhou

**Affiliations:** ^1^College of Computer, National University of Defense Technology, Changsha 410073, China; ^2^LIP 6, Université Pierre et Marie Curie Paris, 75006 Paris, France

## Abstract

To research rumor diffusion in social friend network, based on interests, a dynamic friend network is proposed, which has the characteristics of clustering and community, and a diffusion model is also proposed. With this friend network and rumor diffusion model, based on the zombie-city model, some simulation experiments to analyze the characteristics of rumor diffusion in social friend networks have been conducted. The results show some interesting observations: (1) positive information may evolve to become a rumor through the diffusion process that people may modify the information by word of mouth; (2) with the same average degree, a random social network has a smaller clustering coefficient and is more beneficial for rumor diffusion than the dynamic friend network; (3) a rumor is spread more widely in a social network with a smaller global clustering coefficient than in a social network with a larger global clustering coefficient; and (4) a network with a smaller clustering coefficient has a larger efficiency.

## 1. Introduction

Word-of-mouth communication as a pervasive phenomenon is still a main way for people to exchange information and interact with each other [1]. Social network is the main tunnel for rumor or information diffusion [2, 3]. The status of rumor diffusion is various in different social networks. Currently, many social network models have been proposed such as scale-free [4], small-world [5], random [6], and JGN model [7, 8]. For information or rumor diffusion among friends, most friends have the same or similar interests. For example, for the news that the result of the football match between China versus Thailand was 1 : 5, people in the USA may be not interested in this news, but the Chinese football fans or Thailand football fans are absolutely interested in it. Hence, the interest is a key factor for persons to make friends with others. With mechanisms analysis of the friend network in the real world, we propose a new social network based on interests. People could connect with each other based on interests, and two persons with similar or same interests will more probably connect with each other. Moreover, the strength of a connection between two persons will decay with time until these two persons meet again. In this network model, some parameters could be adjusted to get a corresponding network, and this social network has the characteristics of clustering and community.

Word of mouth is a special mode of rumor diffusion, which is different from the information diffusion in social media [9, 10]. It is impossible to revise a rumor online while forwarding this rumor but people may modify a rumor by the mode of word of mouth in the real world. In order to research the process of rumor diffusion, we propose a new rumor diffusion model—ISS model. In this rumor diffusion model, we define three roles: ignorant, spreader, and stifler. The ignorant role, spreader role; and stifler role mean that people do not get the rumor; people could forward the rumor and people have no interest to forward the rumor, respectively. When an ignorant interacts with a spreader, this ignorant will possibly become a spreader. A spreader may lose the interest for defusing the rumor with time; that is, the interests of a spreader to diffuse rumor decay with time, so a spreader may automatically become a stifler. Meanwhile, if a spreader interacts with another spreader or stifler, this spreader may also become a stifler with a probability. To investigate the rumor diffusion in a dynamic friend network with the ISS diffusion model, we study a case, and we construct an artificial society with the zombie-city model [11, 12]. With simulation experiments, some interesting and valuable conclusions are shown: (1) information without any negative impacts may evolve to become a rumor with negative impacts through the diffusion process; (2) with the same average degree, a random social network is more beneficial for rumor diffusion than the dynamic friend network; (3) a rumor is spread more widely in a social network with a smaller global clustering coefficient than in a social network with a larger global clustering coefficient; (4) a smaller clustering coefficient brings a larger efficiency to a network.

This paper is organized as follows.  Section 2 mainly introduces the dynamic friend network model based on the analysis of the characters of the real social friend network, and some simulation results are presented.  Section 3 proposes a rumor diffusion model, and with this model we study rumor diffusion in a dynamic friend network. Based on the analysis of some simulation experiments, some interesting and valuable conclusions are shown.  Section 4 summarizes the works of this paper, and presents the future works.

## 2. Friend Network Model

### 2.1. Mechanisms of the Dynamic Friend Network

As we know, in the real world every person has limited energy, and people have different capabilities. Hence, different people may have different number of friends. Usually, a person should have similar interests with their friends. The interest is the main factor for people making friends with others. It is impossible for two persons with absolutely different interests to make friends with each other. The probability of two persons with similar interests becoming friends is larger. Therefore, we could summarize some mechanisms for such a friend network and give some basic assumption as follows.

(1) During a limited time, the population is not changing; that is, the total number of people is constant.
(2) Each person has different capabilities, that is, different max number of friends. Max degree distribution of the friend network follows the power-law exponential distribution such as *P*(*k*) ∝ *e* ^−*k*/*m*^. *P*(*k*) means the probability that the max friends number is more than *k*.
(3) Interests will affect whether two persons could become friends. The interests could be abstracted by a serial of binary codes such as 111001. Each binary code indicates an interest, and the binary code means all the interests that we concern. Digit “1” means the person is interested in this corresponding interest. Then, we could calculate the interest distance between different people. This distance denotes the interest differential degree between people. For example, *A* = 111000 and *B* = 101010 describe the interests of two persons, respectively. As shown in the algorithm of interest distance between *A* and *B* is shown in (2), and the distance between *A* and *B* is 2,
  (1) A⊕B=A    B    [1⊕1=0  +1⊕0=1  +1⊕1=0  +1⊕0=0  +1⊕1=1  +1⊕0=0]=2.
(4) *Friendships decay*. If two friends do not see or meet each other for a long time, the friendship may disappear, that is, the friendships decay with time.

### 2.2. Math Model of the Dynamic Friend Network

Here, we use agents to model people in a society. Based on those mechanisms above, we assume that the total number of agents is *N*. And a parameter *p*_*ij*_ denotes the probability of the individual *i* connecting with agent *j* per unit time. Equation (2) presents the connecting probability of the agent *i* and agent*j*,

(2) $$\begin{array}{c} p_{ij}=f\left(d\right)f\left(z_{i}\right)f\left(z_{i}\right). \end{array}$$

Whether two agents *i* and *j* make friends with each other per unit time depends on two factors: (1) the interest distance *d* between these two agents and (2) the number of friends *z*_*i*_ and *z*_*j*_ each agent already has,

(3) $$\begin{array}{c} f\left(d\right)=\frac{p_{1}}{e^{\alpha d}}\;\left(p_{1}\leq 1\right). \end{array}$$

The function *f*(*d*) is presumably larger for smaller *d*, and *d* denotes the interest distance between agent *i* and *j*. When *d* is larger, this function will fall off. Let *I*_*i*_ and *I*_*j*_ denote the interest of agents *i* and *j*, respectively; then *d* = *I*_*i*_ ⊕ *I*_*j*_. In this paper, the length of this binary string is 6; that is, each *I*_*i*_ is made up of 6 binary digits. *α* and *p*_1_ are two adjustable parameters for this function,

(4) $$\begin{array}{c} f_{\left(z_{i}\right)}=\frac{1}{e^{\beta (z_{i}-z_{i}^{*})}+1},\\ f_{\left(z_{j}\right)}=\frac{1}{e^{\beta (z_{j}-z_{j}^{*})}+1}. \end{array}$$

As shown in (4), the function *f*(*z*) is larger for smaller *z* and decreases sharply around the value z* (*z*_i_* and *z*_j_*). *z*_i_* and *z*_j_* indicates the max number of friends that each agent *i* and *j* could have, respectively. And the max degree distribution should follow the exponential distribution as *P*(*k*) ∝ *e* ^−*k*/*m*^. *β* and *m* are two adjustable parameters of the model. Consider

(5) $$\begin{array}{c} S_{ij}=e^{-\kappa \Delta t}, \end{array}$$

where *S*_*ij*_ represents the strength of the friendship between agents *i* and *j*. When two agents *i* and *j* become friends at the moment (or two friends *i* and *j* meet again), the strength *S*_*ij*_ of the connection between these two agents is set to 1. The strength *S*_*ij*_ of connection between agents *i* and *j* will decay with time. Δ*t* indicates the time since these two agents last met, and *κ* is an adjustable parameter for this function. If *S*_*ij*_ is smaller than a threshold *λ*, the friendship between agents *i* and *j* is inactive. When they meet again, this strength *S*_*ij*_ will be set back to 1. Here, the threshold *λ* used in this paper is 0.3.

### 2.3. Results

**Figure 1:**
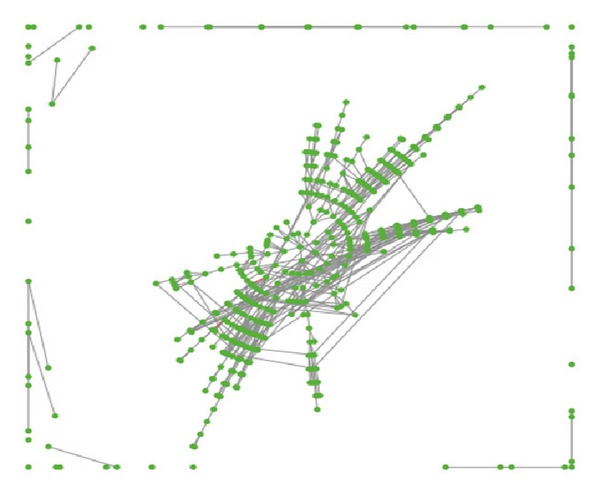
Sample network generated by this dynamic friend network model, where *N* = 500, *α* = 5, *β* = 10, *m* = 5, *κ* = 0.00005, *p*_1_ = 1, and *λ* = 0.3.

In this part, we will study some characteristics of the dynamic friend network.  Figure 1 presents a sample of this dynamic friend network, and we could see the phenomenon of clustering. When the parameter *α* is larger the characteristics of clustering and community are more apparent.

**Figure 2:**
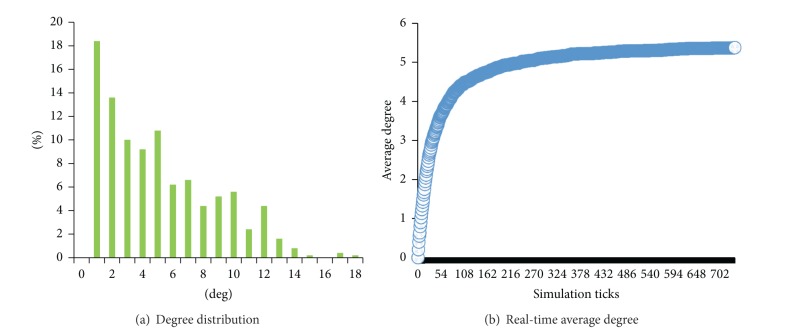
Degree distribution (a) and the average degree (b), where *N* = 500, *α* = 1, *β* = 10, *m* = 5, *κ* = 0.00005, *p*_1_ = 1, and *λ* = 0.3.

As shown in Figure 2, Figure 2(a) depicts the degree distribution and Figure 2(b) presents the real-time average degree. The parameters *β* and *m* determine the degree distribution and the average degree, respectively. If *β* is small, the probability of an agent *i* connecting with more *z*_i_* agentsis large, and vice versa.

The clustering coefficient is a number to denote the degree of clustering. *C*_*i*_ indicates the clustering coefficient of node *i*, and the global clustering coefficient (CC) in (6) is the average number of all these *C*_*i*_, which reflects the global clustering degree of a network. Equation (7) presents the clustering coefficient of node *i*, where *l*_*i*_ indicates the connection (edge) number between all the neighbors of node *i*, and *d*_*i*_ denotes the degree of node *i*. Consider

(6) $$\begin{array}{c} \text{CC}=\frac{1}{N}\underset{i}{\sum }C_{i}, \end{array}$$

(7) $$\begin{array}{c} C_{i}=\frac{2l_{i}}{d_{i}\left(d_{i}-1\right)}. \end{array}$$

**Figure 3:**
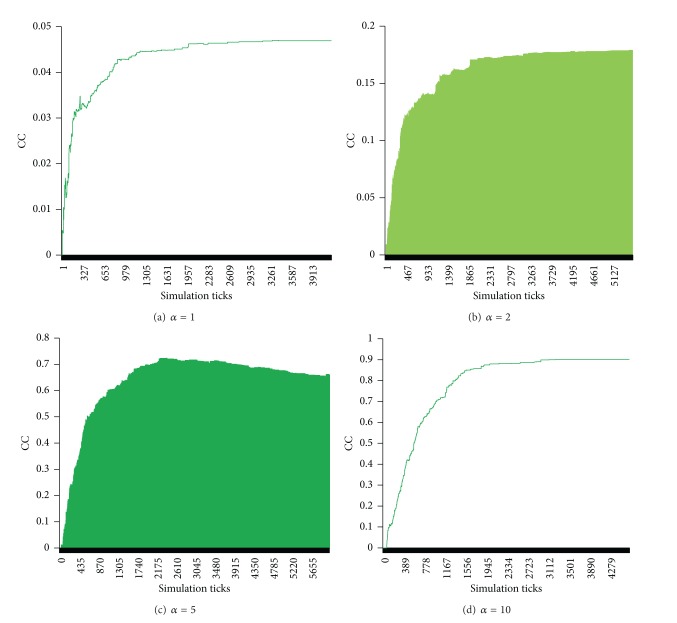
The clustering coefficient with different values of *α*, where *β* = 10, *m* = 5, *κ* = 0.00005, *p*_1_ = 1, and *λ* = 0.3.

The parameter *α* directly affects the value of the global clustering coefficient of the friend network. As shown in Figure 3, with different values of the parameter *α* (1, 2, 5, 10), the global clustering coefficients are different. When *α* is 1, the clustering coefficient is very small and about 0.045, as presented in Figure 3(a). When *α* is set to 2, the global clustering coefficient is sharply increasing and is approximately around 0.17, as shown in Figure 3(b). When the parameter *α* is adjusted to 5, the global coefficient is around 0.65~0.7. When we set this parameter *α* to 10, we could see that the global coefficient is larger and around 0.9. Above all, when the parameter *α* is large, the global clustering coefficient is also large, and vice versa.

**Figure 4:**
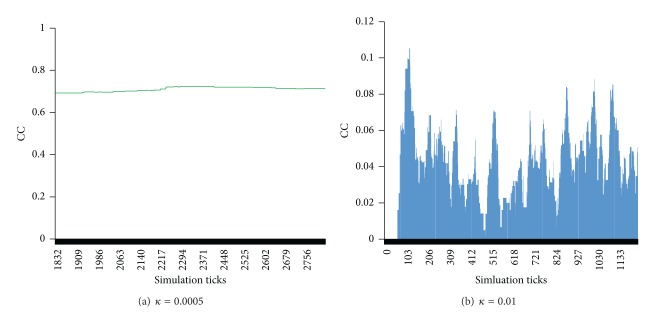
The global clustering coefficient with different values of the parameter *κ*, where *α* = 5, *β* = 10, *m* = 5, *p*_1_ = 1, and *λ* = 0.3.

The parameter *κ* controls the decay speed of the strengths of connections. When the parameter *κ* is small, the decay speed is also slow and the global clustering coefficient is very stable.  Figure 4 shows the global clustering coefficient with different values of the parameter *κ* (0.0005, 0.01) and the same values of other parameters. As presented in Figure 4(a), when the parameter *κ* is 0.00005, for a long time (from the tick 1800 to the tick 2800) the value of the global clustering coefficient is stable around 0.7.  Figure 4(b) presents the value of the global clustering coefficient with *κ* = 0.01, and this value of the global clustering coefficient is fluctuating very sharply.

As these results have shown, we could adjust some parameters such as *α*, *β*, *λ*, *κ*, and *p*_1_ of this friend network model. While the parameter *α* is larger, the global clustering coefficient is bigger. The parameter *β* determines the degree distribution, *m* determines the average degree, and *κ* could affect the stability of the social network. This social friend network has the characteristics of clustering and community.

## 3. Rumor Diffusion in the Social Network

### 3.1. Rumor Diffusion Model

**Figure 5:**
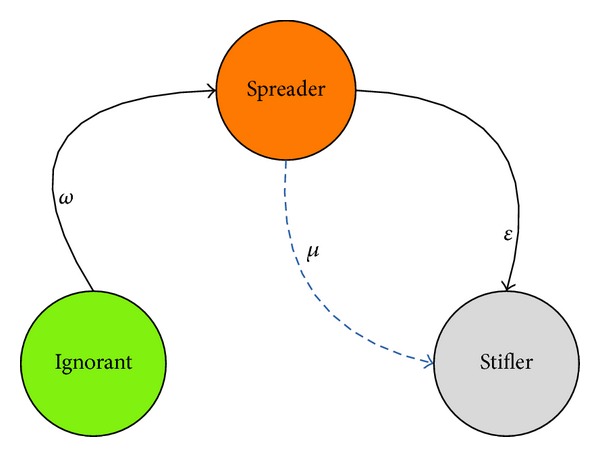
Information diffusion model (ISS).

Although the social network is the main tunnel for rumor diffusion, there is a model to describe the process of rumor diffusion. The process of rumor diffusion is similar to the SIR model for diseases propagation [13–16]. In the rumor diffusion model, people could be in one of these three states, and different states also indicate that people play different roles. There are three roles: ignorant, spread, and stifler. An ignorant means a person who does not know the rumor. A spreader indicates a person who has known the rumor and may spread the rumor to others. A stifler means a person who has known the rumor and lost the interests for this rumor, and this person will not spread the rumor to others [3]. The rumor diffusion model could be seen in Figure 5.

*ω* indicates the infectious probability of an ignorant who becomes a spreader when this ignorant interacts with a spreader; *ε* denotes the immune probability of a spreader who becomes a stifler when the spreader interacts with another spreader or a stifler. The parameter *μ* means that the decay probability of a spreader may automatically become a stifler because the spread could lose their interests on the rumor or they forget to spread the rumor with time. As we know, the parameter *μ* will increase with time, and we could define it as shown in (8). The parameters *p*_0_ and *φ* could control the decay level and the decay speed, respectively. Usually, we define *p*_0_ > 1 and 0 < *φ* < 1,

(8) $$\begin{array}{c} \mu =\frac{1}{1+{p_{0}e}^{-\varphi \Delta t}}. \end{array}$$

Meanwhile, a rumor is evolving with time; that is, a spreader has two choices: to forward this rumor without any revisions to another ignorant or to revise this rumor and spread to another ignorant. Here we define two parameters: *γ* and *τ*. *γ* denotes the probability of a spreader who forward this rumor to another ignorant without any revisions. *τ* indicates the revision rate for this rumor before a spreader forwards this rumor to another ignorant. As seen in (9), a spreader *i* forwards the rumor to an ignorant *j*, and *I*(*i*) means the completeness rate of the rumor in node *i*. If node *m* is the sponsor, then *I*(*m*) = 1,

(9) $$\begin{array}{c} I\left(j\right)=\{\begin{array}{cc} I\left(i\right), & \gamma ,\\ I\left(i\right)*\tau , & 1-\gamma . \end{array} \end{array}$$

### 3.2. Artificial Society Modeling with the Zombie-City Model

In order to study rumor diffusion in a friend network, we should construct an artificial society of a case in the computer world. Based on the zombie-city model [11, 12], we could construct an artificial society from five aspects: agent, role, environment, social network, and rule. And a rumor could be considered as a virus in the zombie-city model.

- Agent: the total number of agents is 500 agents, and agents randomly live in the environment.
- Role: *ignorant *(green color), *spreader* (red color) and *stifler* (gray color).
- Environment: 32 × 32 grids, which is the place where agents live.
- Social network: the dynamic friend network presented in Section 2.
- The rules contain the rules of agents (*R*_*A*_), roles (*R*_*R*_), the environment (*R*_*E*_), and the social network (*R*_*S*_).

*R*_*A*_ *(Rules of Agents).*  (1) At the beginning (at the moment 0) agents randomly live in the environment and all these agents play ignorant. (2) Any agents will randomly interact with their friends per unit time. (3) At a certain time one agent will play the role *spreader *and the completeness rate of the rumor (*I*_*i*_) is 1. (4) The *ignorant* agent may play *spreader* role with a chance of *ω* after interacting with a *spreader* agent, and if a random number (<1) is larger than the parameter *γ*, then this *spreader *agent will forward the rumor with some revision (*τ*) to the *ignorant* agent. If not, this *spreader *agent will forward the rumor without any revision to the *ignorant* agent. (5) Then the *spreader* person may automatically join *stifler* role with a probability of *μ*. (6) A *spreader* agent will become a *stifler* with the probability of *ε* after the agent interacted with a *stifler* or *spreader* agent. These rules could be formally described by the zombie-city model, as shown in Box 1. 

*R*_*R*_ *(Rules of Roles)*. (1) For the *ignorant* role, set the stifler property and the spreader property to false, and set the ignorant property to true.  (2) For the *spreader* role, set the ignorant property and the stifler property to false, and set the spreader property to true. (3) For the *stifler* role, set the spreader property and the ignorant property to false, and set the stifler property to true. These rules could be formally expressed, as shown in Box 2. 

*R*_*S*_ *(Rules of the Social Network)*. (1) Per unit time, each agent *a* randomly selects another agent *b* who has not connected with agent *a*. Calculate the probability *p*_*ij*_ of the connection between these two agents. If this probability *p*_*ij*_ is larger than a random number (<1), create a connection between agents *a* and *b* and *S*_*ij*_ = 1. (2) If any two agents with a connection do not meet, then the strength of each connection will decay per unit time. (3) If the strength of a connection is less than *λ*, then cancel this connection. These rules could be described in formalizations, as shown in Box 3.

### 3.3. Simulations and Experiments

After modeling the artificial society with the zombie-city model, we could implement this case in Netlogo, which is a widely used platform for multiagents system simulations. As we know, there are many parameters to adjust the social network or the rumor diffusion model. However, we just adjust some parameters of the social network to research the impacts of the social network for rumor diffusion, because the social network is the main tunnel for rumor diffusion.

**Figure 6:**
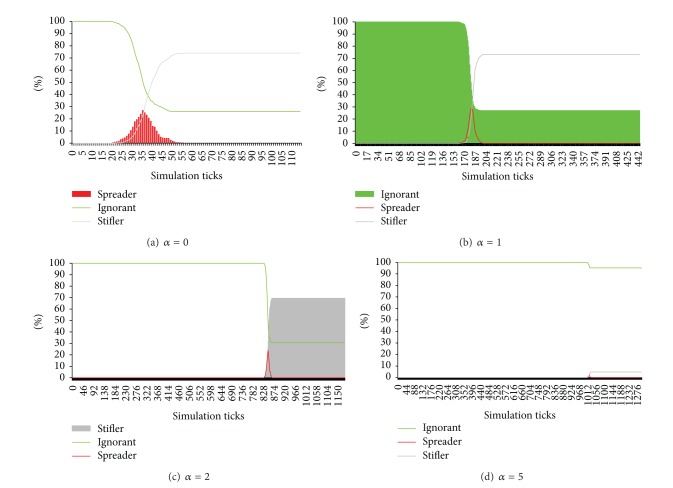
The rumor diffusion status with different values of *α* (0, 1, 2, 5), where *β* = 10, *m* = 5, *p*_1_ = 1, *λ* = 0.3, *κ* = 0.00005, *τ* = 0.95, *γ* = 0.7, *ω* = 0.9, *ε* = 0.3, *p*_0_ = 100, *φ* = 0.1, and *p*_1_ = 1.

As shown in Figure 6, we set these parameters as follows: *β* = 10, *m* = 5, *p*_1_ = 1, *λ* = 0.3, *κ* = 0.00005, *τ* = 0.95, *γ* = 0.7, *ω* = 0.9, *ε* = 0.3, *p*_0_ = 100, *φ* = 0.1, and *p*_1_ = 1, and only adjust the parameter *α*.  Figure 6(a) presents the diffusion status with the parameter *α* = 0. In Figures 6(b), 6(c), and 6(d), the parameter *α* is 1, 2, and 5, respectively. When *α* is 0, the global clustering coefficient is about 0.045 and the percent of agents who have known the rumor (*spreader* and *stifler*) is about 74%. When *α* is 0, the global clustering coefficient is about 0.045 and the percent of agents who have known the rumor (*spreader* and *stifler*) is about 74%. When we set *α* to 1, the global clustering coefficient is around 0.05 and the percent of agents who have known the rumor is about 73%. When *α* is adjusted to 2, the global clustering coefficient is approximately 0.17 and 69.4% agents have known the rumor. When  *α* is set to 5, the global clustering coefficient is about 0.7 and only 4.8% agents have received this rumor. The comparisons of rumor diffusion status with different values of the parameter *α* are shown in Table 1, where the cost time means the time from the tick when the original sponsor created a rumor to the tick when there were no spreaders in the artificial society. With *α* = 5 the cost time is the shortest, because this rumor is only spread in the society with a small scale.

**Figure 7:**
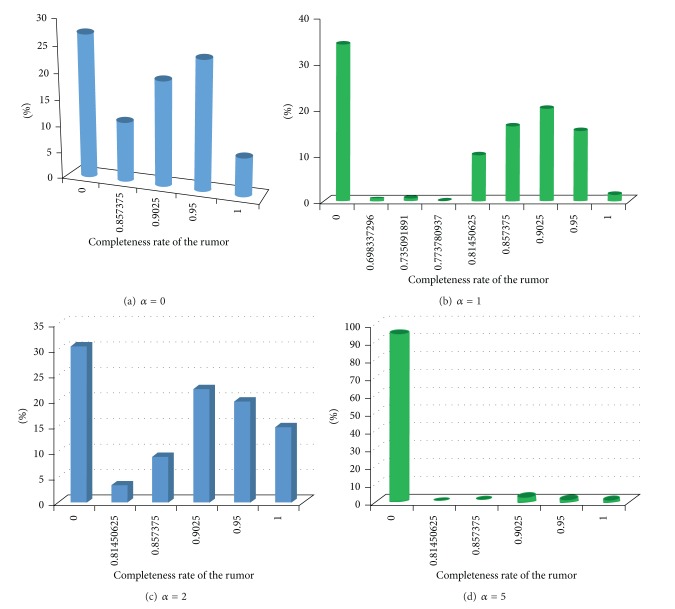
The completeness rate distribution with different values of *α*, where *β* = 10, *m* = 5, *p*_1_ = 1, *λ* = 0.3, *κ* = 0.00005, *τ* = 0.95, *γ* = 0.7, *ω* = 0.9, *ε* = 0.3, *p*_0_ = 100, *φ* = 0.1, and *p*_1_ = 1.

As we know, the rumor diffusion by word of mouth is not similar to the rumor diffusion in social media. People may revise the rumor and then forward another one through the way of word of mouth. Hence, we should research the completeness rate of a rumor that agents have got. The completeness rate of a rumor reflects the revised degree of a rumor.  Figure 7 presents the completeness rate distribution of a rumor with different values of the parameter *α*, and other parameters are defined as follows: *β* = 10, *m* = 5, *p*_1_ = 1, *λ* = 0.3, *κ* = 0.00005, *τ* = 0.95, *γ* = 0.7, *ω* = 0.9, *ε* = 0.3, *p*_0_ = 100, *φ* = 0.1, and *p*_1_ = 1.

For the parameter *α* = 0,1, 2,5, most of the informed agents who have known this rumor received the revised rumor, as shown in Figures 7(a), 7(b), 7(c), and 7(d). Therefore, common news or information may also be revised through the processes of diffusion, and this information may become a rumor in a certain time.

**Figure 8:**
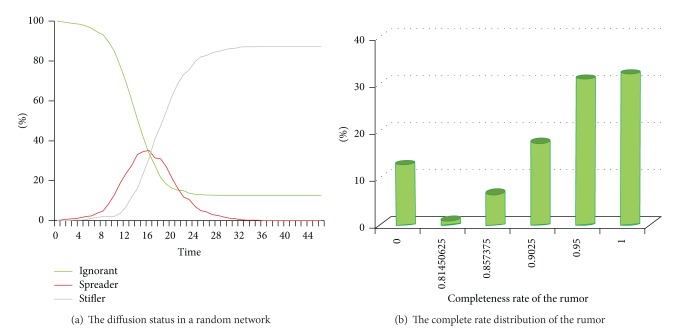
The rumor diffusion status in a random network (average degree of this network is 5).

We could compare the rumor diffusion in this friend network with the rumor diffusion in a random static social network. As shown in Figure 8, the average of this random network is 5, and the global clustering coefficient is about 0.017.  Figure 8(a) presents the rumor diffusion status. 87.4% agents have received this rumor, and the cost time is 27 (from the tick 1 to 28). Similarly, most of the agents who have known this rumor just have got the revised rumor.

### 3.4. Further Discussions

Based on those results above, we could get some interesting conclusions. (1) Common news or information may become a rumor through the complex process of diffusion. (2) With the same average degree, a random social network is more beneficial for rumor diffusion than the dynamic friend network. (3) A rumor is spread more widely in a social network with a smaller global clustering coefficient than in a social network with a larger global clustering coefficient. (4) A smaller clustering coefficient brings a larger efficiency to a network.

Why a random social network is more beneficial for rumor diffusion? As we know, the clustering coefficient of a random network is very small. With a larger clustering coefficient the global connectivity of the network will be decreased. The connectivity will affect the efficiency of a network. The efficiency reflects the transmission capability of a network, which is defined in (10). The parameter *d*_*ij*_ denotes the distance between agents *i* and *j*. If the agent *i* does not reach the agent *j*, the parameter 1/*d*_*ij*_ is 0. If not, the parameter 1/*d*_*ij*_ is 1/*Min*⁡{distance(*i*, *j*)}, that is, the reciprocal of the minimum distance between agents *i* and *j*, as depicted in (11). When the efficiency of a network is large, the effect of information or rumor diffusion is very obvious,

(10) $$\begin{array}{c} E=\frac{1}{N\left(N-1\right)}\underset{i\neq j}{\sum }\frac{1}{d_{ij}}, \end{array}$$

(11) $$\begin{array}{c} \frac{1}{d_{ij}}=\{\begin{array}{cc} \frac{1}{\mathrm{Min}\left\{\text{distance}\left(i,j\right)\right\}}, & \text{if}\;\;\text{node}\;\;i\;\;\text{could}\;\;\text{reach}\;\;\text{node}\;\;j,\\ 0, & \text{otherwise}. \end{array} \end{array}$$

**Figure 9:**
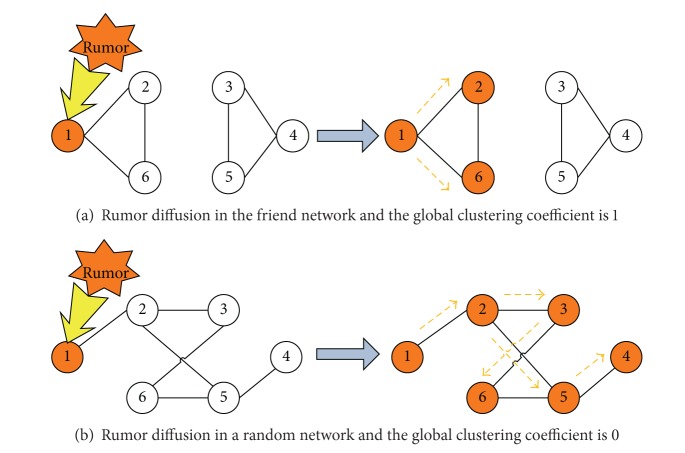
Rumor diffusion in a friend network and a random network.

To clearly understand the differences of rumor diffusion in a friend network and a random network, we could see Figure 9.  Figure 9(a) presents rumor diffusion in the friend network, where the global clustering coefficient (CC) is 1 and the efficiency (*E*) is 0.4. Only three agents could receive this rumor. As shown in Figure 9(b), the global clustering coefficient of this random network is 0 and the efficiency (*E*) is about 0.7. All of these agents could get this rumor. Therefore, a random network with a smaller clustering coefficient and a bigger efficiency is more beneficial for rumor diffusion than the friend network with a larger clustering coefficient and a smaller efficiency.

## 4. Conclusions

The social network is the main tunnel for rumor diffusion, especially for rumor diffusion through word of mouth. Hence, this paper through mechanisms analysis of friendships proposes a dynamic friend network model, and people make friends with each other based on interests. Meanwhile, this model has many parameters for users to adjust. This article also proposes a new rumor diffusion model (*ISS* model). In this model, there are three states: ignorant, spreader, and stifler. The ignorant could receive the rumor and become the spreader only after interacting with a spreader. The spreader may revise the rumor before forwarding to this ignorant. A spreader could become a stifler through two ways: automatically transition (for the interest decay) and after interacting with another spreader or a stifler. A stifler is the one who just knows this rumor but do not have the interest to forward the rumor. To study rumor diffusion in a friend network with different values of these parameters, firstly we construct an artificial society with the zombie-city model and then implement the case in Netlogo. With some simulation experiments, some interesting conclusions could be seen, (1) positive information may evolve to become a rumor through the complex process of diffusion, (2) with the same average degree, a random social network is more beneficial for rumor diffusion than the dynamic friend network, (3) a rumor is spread more widely in a social network with a smaller global clustering coefficient than in a social network with a larger global clustering coefficient, (4) a smaller clustering coefficient brings a larger efficiency to a network.

The future works will contains the following: (1) we will study more cases with the ISS diffusion model and the friend network model, for example, research disease transmission among friends, (2) after perfecting the zombie-city, we will formally reason and analyze the process of rumor diffusion.

## Figures and Tables

**Box 1:**
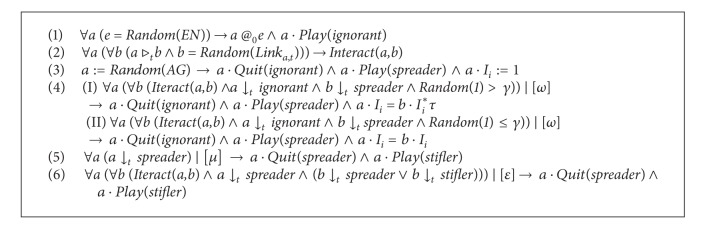
Formalizations of the rules of agents.

**Box 2:**
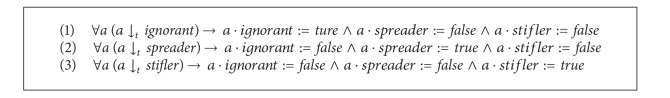
Formalizations of the ignorant role's rules.

**Box 3:**
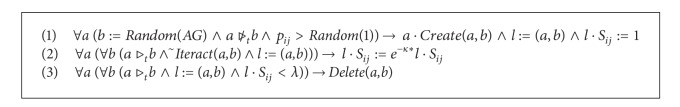
Formalizations of the social network's rules.

**Table 1:** Comparisons of rumor diffusion status with different *α*.

Parameter *α*	Clustering coefficient	Percent of informed agents	Cost time
*α* = 0	0.045	74%	31 (20~51)
*α* = 1	0.05	73%	33 (163~196)
*α* = 2	0.17	69.4%	30 (832~862)
*α* = 5	0.7	4.8%	13 (1011~1024)
